# Pooled safety analysis of lurbinectedin plus irinotecan in patients with advanced solid tumors

**DOI:** 10.1007/s10637-026-01616-0

**Published:** 2026-05-04

**Authors:** Alejandro Falcón, Gregory M. Cote, Javier Molina-Cerrillo, Axel Le Cesne, Pedro Rocha, Bruno Bockorny, David Paez, Javier Martin-Broto, Sant P. Chawla, Sara Martinez, Carmen Kahatt, Vicente Alfaro, Mariano Siguero, Javier Jimenez, Ana Custodio, Vicente Alonso-Orduña, Carlos López, Attila Kollár, Juan José Soto-Castillo, Luis Paz-Ares

**Affiliations:** 1 Medical Oncology Department, Hospital Universitario Virgen del Rocio, Seville, Spain; 2 Medical Oncology Department, Mass General Brigham Cancer Institute, Boston, MA USA; 3 Medical Oncology Department, Hospital Universitario Ramón y Cajal, Madrid, Spain; 4 Medical Oncology Department, Institute Gustave Roussy, Villejuif, France; 5 Medical Oncology Department, Vall d’ Hebrón University Hospital, Barcelona, Spain; 6 Medical Oncology Department, Beth Israel Deaconess Medical Center, Boston, MA USA; 7 Medical Oncology Department, Sant Pau, CIBERER, Hospital de La Santa Creu I, Barcelona, IRSANT PAU Spain; 8 Medical Oncology Department, Fundación Jiménez Díaz University Hospital General de Villalba, and Instituto deInvestigacion Sanitaria Fundacion Jimenez Diaz (IIS/FJDUAM), Madrid, Spain; 9 Sarcoma Oncology Research Center, Santa Monica, USA; 10 Clinical R&D, Pharma Mar, Colmenar Viejo, Madrid, Spain; 11 Medical Oncology Department, Hospital Universitario La Paz, Madrid, Spain; 12 Medical Oncology Department, Hospital Universitario Miguel Servet, Saragossa, Spain; 13 Medical Oncology Department, Hospital Universitario Marqués de Valdecilla, IDIVAL, UNICAN, Santander, Spain; 14 Department of Medical Oncology, Inselspital, Bern University Hospital, University of Bern, Bern, Switzerland; 15 Medical Oncology Department, START Madrid CIOCC, Hospital Universitario HM Sanchinarro, Madrid, Spain; 16 Medical Oncology Department, Hospital Universitario, 12 de Octubre, Madrid, Spain; 17 Servicio de Oncología Médica, Hospital Universitario Virgen del Rocio, Avenida Manuel Siurot, S/N, 41013 Seville, Spain

**Keywords:** Lurbinectedin, Irinotecan, Pooled safety, Phase I, Phase II

## Abstract

Lurbinectedin in combination with irinotecan showed synergistic antitumor activity in preclinical studies. A phase I/II trial (NCT02611024) evaluated this combination in patients with advanced solid tumors. The phase II stage evaluated the antitumor activity of the recommended dose (RD) (lurbinectedin 2.0 mg/m^2^ on Day (D)1 plus irinotecan 75 mg/m^2^ on D1 and D8 q3wk with primary granulocyte colony-stimulating factor prophylaxis) in five tumor-specific cohorts. This pooled analysis describes the safety profile of this combination from 233 patients with different advanced solid tumors treated at the RD. Adverse events (AEs) and laboratory abnormalities were graded using NCI-CTCAE v.4. The most frequent AEs (any grade) related to treatment were fatigue (71% of patients/25% of cycles), diarrhea (62%/17%), nausea (59%/18%), vomiting (35%/7%) and decreased appetite (32%/6%); the most common grade ≥ 3 AEs were fatigue (14%/2%), diarrhea (14%/2%) and febrile neutropenia (9%/1%). The most common grade ≥ 3 laboratory abnormality regardless of relationship was neutropenia (53%). The rate of discontinuation due to treatment-related AEs was 3% (*n* = 7 patients). There was one treatment-related death (0.4%) involving a 63-year-old male patient (0.4%) diagnosed with metastatic NEN who died due to grade 5 staphylococcal bacteremia. In conclusion, this pooled analysis shows a predictable and manageable safety profile for lurbinectedin in combination with irinotecan in patients with advanced solid tumors, with myelosuppression, fatigue and gastrointestinal disorders being the main toxicities observed.

**Trial code:** ClinicalTrials.gov identifier: NCT02611024.

**Supplementary Information:**

The online version contains supplementary material available at 10.1007/s10637-026-01616-0.

## Introduction

Lurbinectedin in combination with irinotecan showed synergistic antitumor activity in preclinical studies [1] and was evaluated in a phase I/II trial (NCT02611024). The phase I stage defined a recommended dose (RD) of lurbinectedin 2.0 mg/m^2^ on Day (D)1 as a 1-h intravenous (i.v.) infusion plus irinotecan 75 mg/m^2^ on D1 and D8 every three weeks (q3wk) as a 90-min i.v. infusion, with primary granulocyte colony-stimulating factor (G-CSF) prophylaxis [2]. The phase II stage evaluated the antitumor activity at the RD in five tumor-specific cohorts: small cell lung cancer (SCLC), soft tissue sarcoma (STS), neuroendocrine neoplasms (NENs), endometrial carcinoma and glioblastoma. Preliminary results showed promising antitumor activity and manageable toxicity in SCLC [3, 4], STS [5], and NENs [6]. Lurbinectedin plus irinotecan is being evaluated in an ongoing randomized phase III study in second-line SCLC (LAGOON; NCT05153239) [7].

This pooled analysis describes the safety profile of lurbinectedin plus irinotecan from patients treated at the RD (lurbinectedin 2.0 mg/m^2^ D1 plus irinotecan 75 mg/m^2^ D1 and D8 q3wk with primary G-CSF prophylaxis) in the phase I/II study.

## Patients and methods

### Patients

This analysis includes safety data from all patients treated with at least one dose of lurbinectedin plus irinotecan at the RD. The trial was conducted at 15 investigational sites in Europe (France, Spain and Switzerland) and the U.S. in compliance with the International Conference on Harmonisation (ICH) Good Clinical Practice guidelines. The protocol was approved by the centers’ Research Ethics Committees. Signed written informed consent was obtained for each patient before any study-specific procedures.

Eligible patients were aged ≥ 18 years; with Eastern Cooperative Oncology Group (ECOG) performance status (PS) score ≤ 1; life expectancy ≥ 3 months; who had been treated with no more than two prior lines of cytotoxic-containing chemotherapy for advanced disease except for SCLC (one prior line of platinum-containing chemotherapy with/without antibodies against programmed cell death protein-1 or programmed death ligand-1) and NENs (one prior line of platinum-based chemotherapy for poorly differentiated neuroendocrine carcinomas, and no more than three prior lines of systemic therapy for neuroendocrine tumors); who had recovered from previous toxicities; and with adequate bone marrow, hepatic, renal and metabolic function.

Patients were excluded if they had been pretreated with lurbinectedin, trabectedin or topoisomerase I inhibitors; had received bone marrow or stem cell transplantation, or radiotherapy in > 35% of bone marrow; were lactating women or were not using effective contraceptives; or had symptomatic brain metastases or leptomeningeal disease, relevant cardiac disease, active infection, any disease interfering with study outcome, or hypersensitivity to lurbinectedin, irinotecan or any formulation component.

Treatment was administered until disease progression, unacceptable toxicity, intercurrent illness precluding study continuation, patient refusal and/or non-compliance with study requirements, treatment delay > 15 days (except if with clear clinical benefit), > 2 dose reductions per drug (irinotecan or lurbinectedin), and treatment-related grade ≥ 3 rhabdomyolysis. Standard antiemetic prophylaxis (dexamethasone 8 mg and ondansetron 8 mg, or equivalent) was given before each infusion. No primary antidiarrheal prophylaxis was administered.

### Study assessments

This pooled safety analysis was based on the assessment of adverse events (AEs) and laboratory abnormalities. Safety was monitored throughout treatment and up to 30 days after the last treatment infusion or start of a new antitumor therapy, whichever occurred first. Any treatment-related AE was followed until recovery to grade ≤ 1 or stabilization of symptoms, or until start of a new antitumor therapy, whichever occurred first. Laboratory assessments were conducted weekly in the first cycle and on Day 1 and Day 8 of subsequent cycles. AEs and laboratory abnormalities were coded using the Medical Dictionary for Regulatory Activities (MedDRA) v.25.0 and graded using the National Cancer Institute Common Terminology Criteria for Adverse Events (NCI-CTCAE) v.4.

### Statistical analysis

Frequency tables were prepared for categorical variables. Continuous variables were described using summary tables with the median and range for each variable. Non-continuous variables were described using frequency tables with counts and percentages. SAS v.9.4 was used for all analyses.

## Results

### Characteristics of patients

The baseline characteristics of the 233 patients treated at the RD and included in this pooled analysis are summarized in Table 1. Of these 233 patients, 230 were treated in the Phase I escalation stage or in cohorts in the Phase II expansion stage (SCLC, n = 113; STS, n = 40; NENs, n = 34; glioblastoma, n = 22; endometrial carcinoma, n = 21), and three patients with other tumor types (epithelial ovarian cancer, n = 2; pancreatic adenocarcinoma, n = 1) were treated only in the Phase I escalation stage. Most patients (n = 126, 54%) were male, White (n = 212, 91%), with ECOG PS score = 1 (n = 156, 67%) and a median age of 60 years (range, 19–77 years). Most patients had locally advanced (n = 65, 28%) or metastatic disease (n = 138, 59%) at diagnosis. Median number of metastatic sites at baseline was 3 (range, 1–8), with 48.2% of patients having < 3 disease sites. Lung (n = 158, 68%), lymph nodes (n = 150, 64%) and liver (n = 78, 34%) were the most common disease sites at baseline. A total of 96 patients (41%) had target lesions with longest diameters summing > 100 mm.

**Table 1:** Baseline characteristics of patients treated with lurbinectedin 2.0 mg/m^2^ 1-h intravenous (i.v.) infusion on D1 plus irinotecan 75 mg/m^2^ 90-min i.v. infusion on D1 and D8 q3wk with primary G-CSF prophylaxis

	**Pooled data** (*n* = 233)
**Gender**	
Male	126 (54)
Female	107 (46)
**Median age, years (range)**	60 (19–77)
≥ 65 years	74 (32)
**Race**	
American Indian or Alaska Native	1 (< 1)
Asian	2 (< 1)
Black or African American	4 (2)
Native Hawaiian or other Pacific Islander	1 (< 1)
White	212 (91.0)
Other ^a^	13 (6)
**ECOG performance status**	
0	77 (33)
1	156 (67)
**Tumor type (cohort)**	
SCLC	113 (49)
STS	40 (17)
NENs	34 (15)
Glioblastoma	22 (9)
Endometrial carcinoma	21 (9)
Other^b^	3 (1)
**Stage at diagnosis**	
Early	30 (13)
Locally advanced	65 (28)
Metastatic	138 (59)
**Sum of target lesions at baseline**	
> 50 mm	189 (81)
> 100 mm	96 (41)
Bulky disease ^c^	92 (40)
**Median no. of tumor sites at baseline (range)**	3 (1–8)
**Most common sites of disease at baseline (≥ 10 patients)**	
Lung	158 (68)
Lymph nodes	150 (64)
Liver	78 (34)
Bone	62 (27)
Central nervous system	55 (24)
Pleura	49 (21)
Adrenal gland	49 (21)
Peritoneal mass/implants	26 (11)
Pancreas	13 (6)
Soft tissue	10 (4)
**Prior surgery**	89 (38)
**Prior radiotherapy**	141 (61)
**Median no. of prior systemic therapy lines (range)**	1 (1–7) ^d^
**Median no. of prior chemotherapy lines (range)**	3 (1–8)
**Median time from diagnosis to first infusion (months)**	11.2 (1.8–220.1)
**Median time from last PD to first infusion (months)**	0.9 (0.1–8.0)
Data are n (%) of patients, except for median (range).	
^a^ Race not reported (*n* = 12) or Latino.	
^b^ Three patients with other type of tumors were treated at the recommended dose only in the Phase I stage: two patients with epithelial ovarian cancer, and one patient with pancreatic adenocarcinoma.	
^c^ Bulky disease is defined as a target lesion measuring at least 50 mm.	
^d^ Three patients with NENs did not receive prior lines of anticancer therapy.	
*ECOG* Eastern Cooperative Oncology Group, *NENs* neuroendocrine neoplasms, *PD* disease progression, *SCLC* small cell lung cancer, *STS* soft tissue sarcoma.	

Prior anticancer therapies comprised surgery (*n* = 89, 38%) and radiotherapy (*n* = 141, 61%). The patients had received a median of one prior line (range, 0–7 lines) and a median of 3 agents (range, 1–8 agents) of systemic anticancer therapies. The prior anticancer agents most frequently administered were platinum compounds (*n* = 163, 70%), etoposide (*n* = 137, 59%), PD-1/PDL-1 inhibitors (*n* = 58, 25%) and anthracyclines (doxorubicin, pegylated liposomal doxorubicin, epirubicin, etc.) (*n* = 45, 19%).

### Treatment exposure

Patients were on treatment for a median of 3.7 months (range, 0.4–61.3 months) (Table 2). The median number of lurbinectedin cycles per patient was 5 (range, 1–83 cycles) and the median relative dose intensity was 95.1% (range, 48.8–105.0%). The median number of irinotecan cycles per patient was 4 (range, 1–83 cycles) and the median relative dose intensity was 79.7% (range, 29.1–104.4%).

**Table 2:** Exposure to lurbinectedin plus irinotecan combination and reasons for treatment discontinuation

	**Lurbinectedin**^**a**^ (*n* = 233)	**Irinotecan** (*n* = 233)
Median no. of cycles administered per patient (range)	5 (1–83)	4 (1–83)
Median time on treatment, months (range)	3.7 (0.4–61.3)	3.7 (0.4–61.3)
Median cumulative dose, mg/m^2^ (range)	8.0 (2.0–120.2)	572.3 (74.2–9163)
Median relative dose intensity, % (range)	95.1 (48.8–105.0)	79.7 (29.1–104.4)
No. of patients who discontinued treatment	233 (100)	
Reasons for treatment discontinuation		
Progressive disease	181 (78)	
Death	14 (6) ^b^	
Non-treatment-related AE	11 (5)	
Investigator’s decision	10 (4)	
Treatment-related AE	7 (3) ^c^	
Patient’s refusal	7 (3)	
Other	3 (1)	

Data are n (%) of patients, except for median (range).

^a^ In case of irinotecan discontinuation, patients could continue treatment with lurbinectedin as a single agent at the approved dose (3.2 mg/m^2^). Eight patients discontinued irinotecan and received single agent lurbinectedin for a total of 48 cycles (median cumulative dose was 18.5 mg/m^2^ and median relative dose intensity was 78.7%). Of these eight patients, three had dose reductions due to treatment-related toxicity, two started treatment at 2.6 mg/m^2^ because they had previously had lurbinectedin dose reductions, and one patient was treated at the dose of 2.0 mg/m^2^ instead of increasing it to 3.2 mg/m^2^ (protocol deviation).

^b^ Of these fourteen deaths, seven were due to disease progression, six were due to non-treatment-related AEs (grade 5 respiratory insufficiency bacteremia, grade 5 pulmonary embolism; grade 5 pneumonia; grade 5 COVID-19 pneumonia; grade 5 respiratory tract infection; and grade 5 septic shock) and one was due to a treatment-related AE (a patient with NEN who died due to grade 5 staphylococcal bacteremia).

^c^ Seven patients (3.0%) discontinued treatment due to treatment-related AEs, which consisted of grade 3 pseudomembranous colitis, grade 2 asthenia, hyperbilirubinemia, grade 2 blood bilirubin and grade 4 transaminase increase, grade 4 septic shock, grade 4 CPK increase, and grade 2 intestinal toxicity.

*AE* Adverse event, *CPK* creatine phosphokinase, *NEN* neuroendocrine neoplasm.

### Adverse events

Pooled incidence of AEs reported among the 233 patients treated at the RD is provided in Table 3 and Table 4.

**Table 3:** Overview of adverse events and support requirement during lurbinectedin plus irinotecan treatment

Patients with:	Pooled data (n = 233)
TEAEs, any grade ^a^	230 (99)
Grade ≥ 3	186 (80)
Treatment-related AEs, any grade ^b^	228 (98)
Grade ≥ 3	160 (69)
Serious TEAEs, any grade	101 (43)
Grade ≥ 3	87 (37)
Serious treatment-related AEs, any grade	48 (21)
Grade ≥ 3	41 (18)
Dose reductions associated with:	
TEAEs	97 (42)
Treatment-related AEs	96 (41)
Deaths associated with:	
TEAEs	14 (6)
Treatment-related AEs	1 (< 1)
Treatment discontinuations associated with:	
TEAEs	21 (9)
Treatment-related AEs	7 (3)
G-CSF therapeutic support	44 (19)
RBC transfusions	53 (23)
Platelet transfusions	7 (3)
Erythropoiesis-stimulating agent use	19 (8)

Data are n (%) of patients.

^a^ TEAEs were defined as any AEs aggravated in severity from baseline or having their onset between the first dose of the study drug and 30 days after the last treatment dose, death or date of further therapy (whichever comes first). AEs related to treatment or with unknown relationship occurring more than 30 days after the last dose were also taken into account as TEAEs.

^b^ Includes AEs related to treatment or with unknown relationship.

AE, adverse event; G-CSF, granulocyte colony-stimulating factor; RBC, red blood cells; TEAE, treatment-emergent adverse event.

**Table 4:** Most common treatment-emergent adverse events, treatment-related adverse events and laboratory abnormalities during lurbinectedin plus irinotecan treatment

	**Pooled data**			
	**Per patient** (*n* = 233)		**Per cycle** (*n* = 1852)	
NCI-CTCAE grade	** ≥ 1**	** ≥ 3**	** ≥ 1**	** ≥ 3**
TEAEs (≥ 10% of patients) ^a^				
Fatigue	79	16	28	2
Diarrhea	65	15	19	3
Nausea	64	4	20	< 1
Decreased appetite	41	3	8	< 1
Vomiting	40	5	8	< 1
Constipation	28	0	5	0
Dyspnea	19	< 1	3	< 1
Pyrexia	19	< 1	3	< 1
Weight decreased	18	< 1	3	< 1
Back pain	15	< 1	3	< 1
Abdominal pain	13	< 1	3	< 1
Cough	13	0	2	0
Headache	13	< 1	2	< 1
Arthralgia	12	< 1	2	< 1
Peripheral edema	11	< 1	2	< 1
Treatment-related AEs (≥ 5% of patients) ^b^				
Fatigue	71	14	25	2
Diarrhea	62	14	17	2
Nausea	59	4	18	< 1
Vomiting	35	5	7	< 1
Constipation	10	0	2	0
Decreased appetite	32	2	6	< 1
Febrile neutropenia	9	9	1	1
Abdominal pain	6	0	1	0
Mucosal inflammation	6	0	1	0
Weight decreased	6	0	< 1	0
Hematological laboratory abnormalities				
Anemia	95	28	87	6
Leukopenia	84	44	48	9
Neutropenia	75	53	34	11
Thrombocytopenia	59	11	26	2
Biochemical laboratory abnormalities				
Creatinine increased	84	2	59	< 1
AP increased	73	4	53	< 1
ALT increased	59	8	26	1
AST increased	50	7	16	< 1
Bilirubin increased	18	2	3	< 1
CPK increased	11	1	2	< 1

Data are % of patients and % of cycles.

Hematological and biochemical abnormalities are shown regardless of relationship to treatment.

^a^ TEAEs were defined as any AEs aggravated in severity from baseline or having their onset between the first dose of the study drug and 30 days after the last treatment dose, death or date of further therapy (whichever comes first). AEs related to treatment or with unknown relationship occurring more than 30 days after the last dose were also taken into account as TEAEs.

^b^ Includes AEs related to treatment or with unknown relationship.

*AE* adverse event, *ALT* alanine aminotransferase, *AP* alkaline phosphatase, *AST* aspartate aminotransferase, *CPK* creatine phosphokinase, *NCI-CTCAE* National Cancer Institute Common Terminology Criteria for Adverse Events, *TEAEs* treatment-emergent adverse events

The most common treatment-emergent AEs (TEAEs) of any grade were fatigue (79% of patients/28% of cycles), gastrointestinal disorders (diarrhea [65%/19%], nausea [64%/20%], vomiting [40%/8%] and constipation [28%/5%]), and decreased appetite (41%/8%) (Table 4). Most of these TEAEs were grade 1 or 2; the most common grade ≥ 3 TEAEs comprised fatigue (16%/2%), diarrhea (15%/3%), febrile neutropenia (9%/1%), vomiting (5%/< 1%), nausea (3%/< 1%) and decreased appetite (3%/< 1%).

The most frequent AEs of any grade that were related to treatment (or with unknown relationship) were fatigue (71% of patients/25% of cycles), gastrointestinal disorders (diarrhea [62%/17%], nausea [59%/18%], vomiting [35%/7%], and constipation [10%/2%]), and decreased appetite (32%/6%) (Table 4). The most common of these events to reach grade ≥ 3 were fatigue (14%/2%), diarrhea (14%/2%), febrile neutropenia (9%/1%), vomiting (5%/< 1%), nausea (4%/< 1%) and decreased appetite (2%/< 1%).

Use of loperamide, hydration or atropine was allowed in case of diarrhea. Antipropulsives (loperamide/atropine) were given to 72 patients (31%): therapeutic in 53 patients (23%), prophylactic in 22 patients (9%), and both in three patients (1%). Thirty-three patients (14%) had grade ≥ 3 diarrhea related to the study treatment (Table 4). Fourteen of these 33 patients required hospitalization although in several of these cases other events, some of them related to the disease under study, were also present and justified the admission to hospital. Irinotecan dose was omitted or reduced in nine of these 14 patients. All these cases were resolved except for one patient with STS who died before recovering due to a hemorrhagic shock from a small bowel metastasis. Grade 1/2 dehydration was reported in two patients (0.9%); both patients had concomitant grade 2 diarrhea but they did not require hospitalization or treatment modification.

### Laboratory abnormalities

The most common grade ≥ 3 laboratory abnormalities regardless of relationship were hematological (Table 4). Grade ≥ 3 neutropenia (53% of patients/11% of cycles; grade 4 in 30% of patients/5% of cycles) lasted a median of 5 days (range, 0–46 days) and was mostly afebrile; severe treatment-emergent febrile neutropenia was observed in 9% of patients. Grade ≥ 3 anemia (28% of patients/6% of cycles; grade 4 in 0%) occurred mostly among patients who already had anemia at baseline. Grade ≥ 3 thrombocytopenia (11% of patients/2% of cycles; grade 4 in 4% of patients and < 1% of cycles) lasted a median of 6 days (range, 1–14 days) and was not associated with bleeding episodes. Overall, 44 patients (19%) required G-CSF therapeutic support additional to the G-CSF primary prophylaxis, 53 patients (23%) received red blood cell transfusions, seven (3%) received platelet transfusions, and 19 (8%) received erythropoiesis-stimulating agents during treatment (Table 3).

Grade ≥ 3 transaminase increases were the most common severe laboratory biochemical abnormalities regardless of the relationship (Table 4). Grade ≥ 3 alanine aminotransferase (ALT) increases (8% of patients/1% of cycles) lasted a median of 3.5 days (range, 1–21 days). Grade ≥ 3 aspartate aminotransferase (AST) increases (7% of patients/< 1% of cycles) lasted a median of 3 days (range, 1–28 days).

### Serious adverse events, dose reductions, treatment discontinuations and deaths

Serious treatment-related AEs (any grade) were reported in 48 patients (21%) (Table 3); the most common were gastrointestinal disorders (mostly diarrhea [*n* = 22 cycles, 1%] and vomiting [*n* = 4 cycles, < 1%]), hematological disorders (febrile neutropenia [*n* = 17 cycles, < 1%], neutropenia [*n* = 4 cycles, < 1%], thrombocytopenia [*n* = 3 cycles, < 1%] and anemia [*n* = 1 cycle, < 1%]), and infections and infestations (mostly septic shock [*n* = 4 cycles, < 1%]).

Dose reductions due to treatment-related AEs occurred in 96 patients (41%) (Table 3). Lurbinectedin dose reductions were mostly due to hematological toxicity (neutropenia alone [*n* = 40 cycles] as the most common cause). Irinotecan dose reductions were mostly due to non-hematological toxicity (mostly diarrhea alone [*n* = 26 cycles]).

Most treatment discontinuations (*n* = 181, 78%) were due to disease progression (Table 2). The rate of treatment discontinuation due to any grade treatment-related AEs was 3% (*n* = 7 patients); these AEs consisted of grade 3 pseudomembranous colitis, grade 2 asthenia, grade 4 hyperbilirubinemia, grade 2 blood bilirubin and grade 4 transaminase increase, grade 4 septic shock, grade 4 CPK increase, and grade 2 intestinal toxicity (Table 3).

Death related to treatment occurred in one patient (0.4%); this was a 63-year-old male diagnosed with metastatic NEN who received one treatment cycle and on Day 8 was hospitalized with grade 4 febrile neutropenia and grade 5 staphylococcal bacteremia. Despite antibiotic therapy, the patient died on Day 24 due to the staphylococcal bacteremia.

## Discussion

**Fig. 1:**
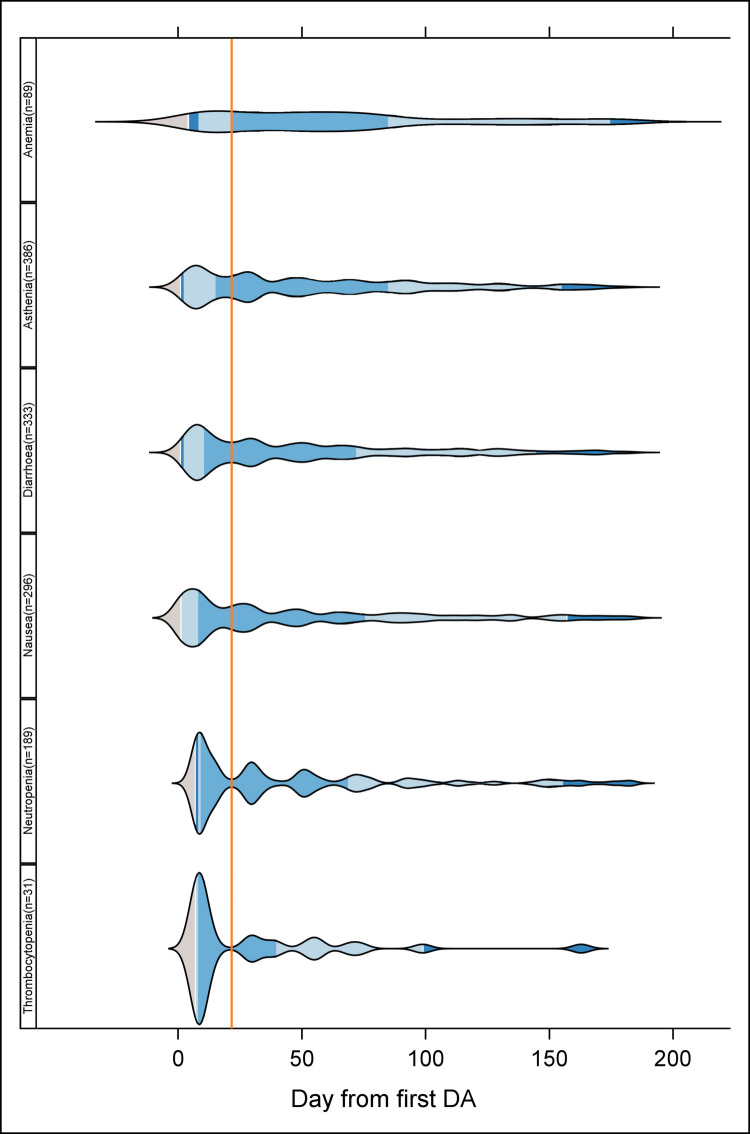
Violin box plot showing the time to onset of the most common adverse events related to treatment and hematological abnormalities regardless of relationship from the first dose administration (DA) of lurbinectedin/irinotecan. Within each plot, the grey area shows the time between the first DA and the first appearance of the event, while the interquartile range is indicated with different shades of blue. The breadth of the plots indicates the patient distribution at any particular time point, whereas the height is proportional to the incidence rate of each event/abnormality. The end of Cycle 1 is shown with an orange line. n, number of event episodes included in each plot

The combination of lurbinectedin 2.0 mg/m^2^ D1 plus irinotecan 75 mg/m^2^ D1 and D8 q3wk with primary G-CSF prophylaxis was well tolerated and showed a predictable and manageable safety profile. Few patients (7/233, 3%) discontinued the study treatment due to treatment-related AEs. The most common treatment-related AEs were fatigue, gastrointestinal disorders (diarrhea, nausea and vomiting) and decreased appetite; these events were mostly grade 1/2 and rarely had any effects on treatment. Proactive management of diarrhea with loperamide and close monitoring for dehydration is essential for maintaining the RD. The time to onset and time to resolution of most common treatment-related AEs and hematological abnormalities regardless of relationship is shown in Fig. 1 and Supplementary Fig. 1, respectively; these events appeared shortly after first dose administration, but they decreased in severity over time.

Anemia was the most common hematological abnormality (all grades), while neutropenia was the most common grade 3/4 hematological abnormality. Grade 3/4 neutropenia was found in 53% of patients and reached grade 4 in 30% but was not cumulative over time and was managed with delays, irinotecan dose omissions and dose reductions. The grade 3/4 neutropenia rate of 53% corresponds to laboratory abnormalities but decreased to 29% when reported as a grade 3/4 AE; therefore, about half of severe neutrophils decreases where a laboratory finding with no symptoms. Time to onset of neutropenia was higher in the first cycle and decreased over time (Fig. 1). The median duration of grade 3/4 neutropenia was the same in Cycle 1 (5 days) that in all cycles. Neutropenia reported with lurbinectedin monotherapy without G-CSF primary prophylaxis is lowest (41%) [8] and combinations of lurbinectedin with other anticancer agents with known hematological toxicities (irinotecan in LAGOON trial [7] or doxorubicin in SaLuDo trial [9]) are given with primary G-CSF prophylaxis to prevent the additive effect on myelosuppression. Grade 3/4 thrombocytopenia was found in 10.7% of patients and was also transient and mostly resulted in irinotecan dose omissions. Few biochemical abnormalities reached grade 3/4, with the most common being transaminase increases.

Despite the most common treatment-related AEs in all cohorts were fatigue, gastrointestinal disorders and decreased appetite, the frequencies of severe AEs were higher in the SCLC and endometrial carcinoma cohorts likely due to higher exposure. Furthermore, some tumor-specific safety signals should be considered; likely due to the nature of the disease, which affects mainly the gastrointestinal system, severe biochemical abnormalities reported in the NEN cohort showed the highest frequencies (Supplementary Table 2).

The safety profile of lurbinectedin plus irinotecan at the RD in this phase I/II study was compared with pooled data from 554 patients treated with lurbinectedin 3.2 mg/m^2^ q3wk in one phase II trial (relapsed SCLC patients) and one phase III trial (relapsed ovarian cancer patients) [8]. Treatment-related AEs rate (both all grades and grade ≥ 3) was higher with lurbinectedin plus irinotecan than with single-agent lurbinectedin (Supplementary Table 1). As a result, more patients treated with lurbinectedin plus irinotecan required dose reduction due to treatment-related AEs than patients treated with single-agent lurbinectedin (41% *vs.* 21%). However, the percentage of patients discontinuing treatment due to treatment-related AEs was the same (3%) with lurbinectedin plus irinotecan and with single-agent lurbinectedin. Furthermore, one treatment-related death occurred with lurbinectedin plus irinotecan (0.4%) at the RD, whereas seven patients (1%) treated with single-agent lurbinectedin died due to AEs related to treatment or with unknown relationship (Supplementary Table 1).

Compared with single-agent lurbinectedin, patients treated with lurbinectedin plus irinotecan showed higher incidences of treatment-related fatigue (71% *vs.* 53%), diarrhea (62% *vs.* 13%), decreased appetite (32% *vs.* 17%) and vomiting (35% *vs.* 25%) (Supplementary Table 1). Severe hematological abnormalities were more common with lurbinectedin plus irinotecan than with lurbinectedin alone (neutropenia 53% *vs.* 41%; leukopenia 44% *vs.* 30%; anemia 28% *vs.* 17%) (Supplementary Table 1). These findings agree with the results of phase II/III studies conducted with single-agent irinotecan in patients with metastatic colorectal cancer [10–13]. In these studies, fatigue, gastrointestinal disorders (mainly diarrhea, nausea and vomiting) and neutropenia were the most clinically relevant AEs related to irinotecan administered weekly or every three weeks.

In conclusion, lurbinectedin 2.0 mg/m^2^ as an i.v. infusion of one hour on D1 plus irinotecan 75 mg/m^2^ as an i.v. infusion over 90 min on D 1 and D8 Day 1 q3wk with primary G-CSF prophylaxis showed a predictable and manageable safety profile in this pooled analysis, with myelosuppression, fatigue and gastrointestinal disorders being the main toxicities observed. Few patients (3%) stopped receiving the combination due to treatment-related events and only one treatment-related death occurred, thereby confirming the tolerability of the combination.

## Supplementary Information

Below is the link to the electronic supplementary material.

ESM 1 (PDF 276 KB)

## Data Availability

The datasets generated or analyzed during the current study are available from the corresponding author on reasonable request.
