# Supplementary material for: Pooled safety analysis of lurbinectedin plus irinotecan in patients with advanced solid tumors
Source: Invest New Drugs. 2026 May 4;44(3):274–83. doi: 10.1007/s10637-026-01616-0 (PMC13486034; doi:10.1007/s10637-026-01616-0)

Supplementary information

Article title: Pooled Safety Analysis of Lurbinectedin plus Irinotecan in Patients with Advanced Solid Tumors

Journal name: Inv New Drugs

Author names: Alejandro Falcón[1] · Gregory M. Cote[2] · Javier Molina-Cerrillo[3] · Axel Le Cesne[4] · Pedro Rocha[5] · Bruno Bockorny[6] · David Paez[7] · Javier Martin-Broto[8] · Sant P. Chawla[9] · Sara Martinez[10] · Carmen Kahatt[10] · Vicente Alfaro[10] · Mariano Siguero[10] · Javier Jimenez[10] · Ana Custodio[11] · Vicente Alonso-Orduña[12] · Carlos López[13] · Attila Kollár[14] · Juan José Soto-Castillo[15] · Luis Paz-Ares[16]

Author affiliation:

1. Medical Oncology Department, Hospital Universitario Virgen del Rocio, Sevilla, Spain.
2. Medical Oncology Department, Mass General Brigham Cancer Institute, Boston, MA, USA.
3. Medical Oncology Department, Hospital Universitario Ramón y Cajal, Madrid, Spain.
4. Medical Oncology Department, Institute Gustave Roussy, Villejuif, France.
5. Medical Oncology Department, Vall d'Hebrón University Hospital, Barcelona, Spain.
6. Medical Oncology Department, Beth Israel Deaconess Medical Center, Boston, MA, USA.
7. Medical Oncology Department, Hospital de La Santa Creu I Sant Pau, IR SANT PAU, CIBERER, Barcelona, Spain.
8. Medical Oncology Department, Fundación Jiménez Díaz University Hospital General de Villalba, and Instituto de Investigacion Sanitaria Fundacion Jimenez Diaz (IIS/FJD; UAM), Madrid, Spain.
9. Sarcoma Oncology Research Center, Santa Monica, USA.
10. Clinical R&D, Pharma Mar, Colmenar Viejo, Madrid, Spain
11. Medical Oncology Department, Hospital Universitario La Paz, Madrid, Spain.
12. Medical Oncology Department, Hospital Universitario Miguel Servet, Zaragoza, Spain.
13. Medical Oncology Department, Hospital Universitario Marqués de Valdecilla, IDIVAL, UNICAN, Santander, Spain.
14. Department of Medical Oncology, Inselspital, Bern University Hospital, University of Bern, Bern, Switzerland.
15. Medical Oncology Department, START Madrid CIOCC, Hospital Universitario HM Sanchinarro, Madrid, Spain.
16. Medical Oncology Department, Hospital Universitario 12 de Octubre, Madrid, Spain.

Corresponding author: Alejandro Falcón, M.D., Ph.D.
E-mail: afalconglez@gmail.com

Supplementary Table 1. Summary of adverse events and laboratory abnormalities reported with irinotecan/lurbinectedin at the recommended dose (RD) in this phase I/II study and with single agent lurbinectedin in previous phase II/III trials.

	Lurbinectedin 2.0 mg/m^2 + irinotecan 75 mg/m^2 Phase I/II trial [a] (n=233)		Lurbinectedin 3.2 mg/m^2 Phase II/III trials [1] (n=554)	
**Patients with:**	**n (%)**		**n (%)**	
**At least one TEAE regardless of relationship**	230 (99)		545 (98)	
**Any grade ≥3 TEAE**	186 (80)		353 (64)	
**Any SAE**	101 (43)		226 (41)	
**TEAEs leading to dose reduction**	97 (42)		117 (21)	
**TEAEs leading to death**	14 (6)		20 (4)	
**TEAEs leading to treatment discontinuation**	21 (9)		52 (9)	
**Any treatment-related (or unknown) AE**	228 (98)		494 (89)	
**Any grade ≥3 treatment-related (or unknown) AE**	160 (69)		244 (44)	
**Any treatment-related (or unknown) SAE**	48 (21)		89 (16)	
**Treatment-related (or unknown) AEs leading to dose reduction**	96 (41)		115 (21)	
**Treatment-related (or unknown) AEs leading to death**	1 (<1)		7 (1)	
**Treatment-related (or unknown) AEs leading to treatment discontinuation**	7 (3)		18 (3)	
**Treatment-related AEs (or with unknown relationship)**	**Grade ≥1**	**Grade ≥3**	**Grade ≥1**	**Grade ≥3**
Fatigue	71	14	53	7
Diarrhea	62	14	13	<1
Nausea	59	4	51	3
Vomiting	35	5	25	3
Decreased appetite	32	2	17	0
Constipation	10	0	17	<1
Febrile neutropenia [b]	9	9	7	7
**Hematological laboratory abnormalities (regardless of relationship)**	**Grade ≥1**	**Grade ≥3**	**Grade ≥1**	**Grade ≥3**
Anemia	95	28	92	17
Leukopenia	84	44	73	30
Neutropenia [b]	75	53	64	41
Thrombocytopenia	59	11	49	10
**Biochemical laboratory abnormalities (regardless of relationship)**	**Grade ≥1**	**Grade ≥3**	**Grade ≥1**	**Grade ≥3**
Creatinine increased	84	2	84	2
AP increased	73	4	46	5
ALT increased	59	8	66	6
AST increased	50	7	53	3
Bilirubin increased	18	2	12	2

Data shown are % of patients.

[a] ClinicalTrials.gov identifier: NCT02611024.

[b] Primary prophylaxis with G-CSF was compulsory in this phase I/II study and was not allowed in the phase II/III trials with single-agent lurbinectedin 3.2 mg/m^2 q3wk.

AE, adverse event; ALT, alanine aminotransferase; AP, alkaline phosphatase; AST, aspartate aminotransferase; G-CSF, granulocyte colony-stimulating factor; q3wk, every three weeks; SAE, serious adverse event; TEAE, treatment-emergent adverse event.

References

1. Leary A, Oaknin A, Trigo JM, Moreno V, Delord JP, Boni V, Brana I, Fernandez C, Kahatt C, Nieto A, Cullell-Young M, Zeaiter A, Subbiah V (2023) Pooled Safety Analysis of Single-Agent Lurbinectedin in Patients With Advanced Solid Tumours. Eur J Cancer 192:113259.

Supplementary Table 2. Most common grade 3/4 treatment-related adverse events and laboratory abnormalities during lurbinectedin plus irinotecan treatment (worst grade per patient and cycle). Data are shown separately by cohort.

	SCLC		STS		NENs		Glioblastoma		Endometrial carcinoma	
	Per patient (n=113)	Per cycle (n=924)	Per patient (n=40)	Per cycle (n=266)	Per patient (n=34)	Per cycle (n=271)	Per patient (n=22)	Per cycle (n=59)	Per patient (n=21)	Per cycle (n=326)
**Median (range) of cycles per patient: Lurbinectedin/ Irinotecan**	7.0 (1-44) / 6.0 (1-44)		4.5 (1-22) / 4.0 (1-22)		3.5 (1-47) / 3.5 (1-47)		2.0 (1.7) / 2.0 (1.7)		4.0 (2-83) / 4.0 (2-83)	
**TEAEs (≥10% of patients) [a]**										
**Diarrhea**	19	3	8	2	12	2	0	0	29	3
**Fatigue**	22	4	10	2	9	1	0	0	19	2
**Febrile neutropenia**	10	1	8	1	6	<1	14	5	10	<1
**Nausea**	4	<1	0	0	6	<1	0	0	14	1
**Treatment-related AEs (≥5% of patients) [b]**										
**Diarrhea**	19	3	8	2	9	1	0	0	29	3
**Fatigue**	19	3	8	1	9	1	0	0	19	2
**Febrile neutropenia**	10	1	8	1	6	<1	14	5	10	<1
**Nausea**	4	<1	0	0	6	<1	0	0	14	1
**Vomiting**	6	1	3	<1	6	<1	0	0	5	<1
**Hematological laboratory abnormalities**										
**Anemia**	30	6	30	7	24	5	5	2	43	6
**Leukopenia**	46	10	55	14	32	6	50	19	33	4
**Neutropenia**	56	13	65	13	47	9	41	15	48	7
**Thrombocytopenia**	12	2	13	2	12	2	9	3	5	<1
**Biochemical laboratory abnormalities**										
**ALT increased**	9	1	5	<1	15	2	9	5	0	0
**AP increased**	3	<1	3	<1	15	3	0	0	0	0
**AST increased**	8	1	3	<1	18	2	5	2	0	0
**Bilirubin increased**	0	0	0	0	15	2	0	0	0	0
**CPK increased**	2	<1	3	<1	0	0	0	0	0	0
**Creatinine increased**	1	<1	3	<1	6	<1	0	0	0	0

Data are % of patients and % of cycles.
Hematological and biochemical abnormalities are shown regardless of relationship to treatment.
[a] TEAEs were defined as any AEs aggravated in severity from baseline or having their onset between the first dose of the study drug and 30 days after the last treatment dose, death or date of further therapy (whichever comes first). AEs related to treatment or with unknown relationship occurring more than 30 days after the last dose were also taken into account as TEAEs.
[b] Includes AEs related to treatment or with unknown relationship.

	SCLC	STS	NENs	Glioblastoma	Endometrial carcinoma

AE, adverse event; ALT, alanine aminotransferase; AP, alkaline phosphatase; AST, aspartate aminotransferase; CPK, creatine phosphokinase; NCI-CTCAE, National Cancer Institute Common Terminology Criteria for Adverse Events; NENs, neuroendocrine neoplasms; SCLC, small cell lung cancer; STS, soft tissue sarcoma; TEAEs, treatment-emergent adverse events.

Supplementary Figure 1. Violin box plot showing the time to resolution of the most common adverse events related to treatment and hematological abnormalities regardless of relationship from the first dose administration (DA) of lurbinectedin/irinotecan. Within each plot, the grey area shows the time between the first DA and the first resolution of the event, while the interquartile range is indicated with different shades of blue. The breadth of the plots indicates the patient distribution at any particular time point, whereas the height is proportional to the incidence rate of each event/abnormality. The end of Cycle 1 is shown with an orange line. n, number of event episodes included in each plot.

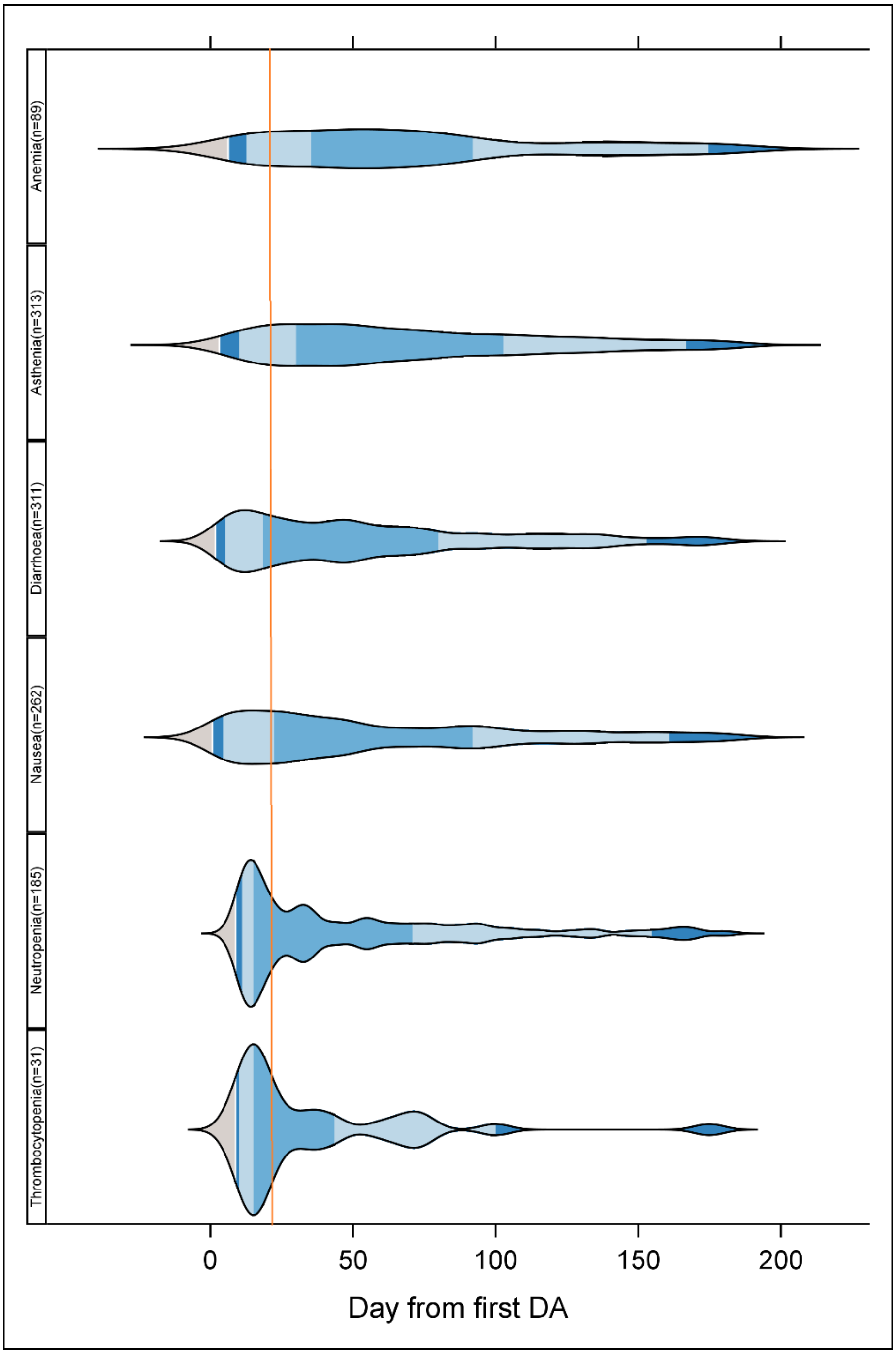

Supplement: Supplementary file 1 [file 10637_2026_1616_MOESM1_ESM.pdf]
